# REFRAMING INTEGRATION OF GERIATRICS PRINCIPLES: A STEALTH APPROACH

**DOI:** 10.1093/geroni/igae098.1085

**Published:** 2024-12-31

**Authors:** Christina Cauble, Benjamin Rosenstein, Alissa Cooney

**Affiliations:** University of Minnesota Medical School, Minneapolis, Minnesota, United States; University of Minnesota, Department of Family Medicine and Community Health, Minneapolis, Minnesota, United States; UT Health San Antonio, San Antonio, Texas, United States

## Abstract

Most healthcare professionals do not receive specific training in geriatrics despite caring for an overwhelmingly large proportion of older adults throughout their careers. While educational programs that feed into geriatrics as a specialty are sometimes more open to adapting curricula to incorporate geriatrics principles, utilizing a stealth geriatrics education approach can help to successfully integrate geriatric principles for a wider range of learning experiences. This session explores how geriatrics educators can integrate basic geriatrics principles into training and learning experiences in a variety of settings for learners who may not intend to specialize in geriatrics. Many health education programs don’t teach robustly on geriatrics principles and struggle to fit additional dedicated time for geriatrics within their curriculum, even though the majority of healthcare workers will at some point provide care to older adults. This segment will examine successful examples of stealthy integration of geriatrics principles into health education for learners regardless of their expectation to further specialize in geriatrics. Educational examples of integrating stealth geriatrics principles include; ‘geriatricizing’ a family medicine case study, a dementia didactic experience for medical students, and an emergency department scenario geared towards integrating stealth geriatrics into bedside nursing, resident, and staff education. Attendees will be encouraged to reframe ways to continue incorporating stealth geriatrics into other educational settings.

